# Improving preventive health care in Aboriginal and Torres Strait Islander primary care settings

**DOI:** 10.1186/s12992-017-0267-z

**Published:** 2017-07-14

**Authors:** Jodie Bailie, Veronica Matthews, Alison Laycock, Rosalie Schultz, Christopher P. Burgess, David Peiris, Sarah Larkins, Ross Bailie

**Affiliations:** 1 The University of Sydney, University Centre for Rural Health - North Coast, Lismore, NSW Australia; 2 Charles Darwin University, Menzies School of Health Research, Darwin, NT Australia; 3 Flinders University, Centre for Remote Health, Alice Springs, NT Australia; 4 Department of Health, Northern Territory Government, Darwin, NT Australia; 5 The University of New South Wales, George Institute for Global Health, Sydney, NSW Australia; 6 James Cook University, College of Medicine and Dentistry, Townsville, QLD Australia

**Keywords:** Preventive health care, Primary health care, Aboriginal and Torres Strait Islander health

## Abstract

**Background:**

Like other colonised populations, Indigenous Australians experience poorer health outcomes than non-Indigenous Australians. Preventable chronic disease is the largest contributor to the health differential between Indigenous and non-Indigenous Australians, but recommended best-practice preventive care is not consistently provided to Indigenous Australians. Significant improvement in health care delivery could be achieved through identifying and minimising evidence-practice gaps. Our objective was to use clinical audit data to create a framework of the priority evidence-practice gaps, strategies to address them, and drivers to support these strategies in the delivery of recommended preventive care.

**Methods:**

De-identified preventive health clinical audit data from 137 primary health care (PHC) centres in five jurisdictions were analysed (*n* = 17,108 audited records of well adults with no documented major chronic disease; 367 system assessments; 2005–2014), together with stakeholder survey data relating to interpretation of these data, using a mixed-methods approach (*n* = 152 responses collated in 2015–16). Stakeholders surveyed included clinicians, managers, policy officers, continuous quality improvement (CQI) facilitators and academics. Priority evidence-practice gaps and associated barriers, enablers and strategies to address the gaps were identified and reported back through two-stages of consultation. Further analysis and interpretation of these data were used to develop a framework of strategies and drivers for health service improvement.

**Results:**

Stakeholder identified priorities were: following-up abnormal test results; completing cardiovascular risk assessments; timely recording of results; recording enquiries about living conditions, family relationships and substance use; providing support for clients identified with emotional wellbeing risk; enhancing systems to enable team function and continuity of care. Drivers identified for improving care in these areas included: strong Indigenous participation in the PHC service; appropriate team structure and function to support preventive care; meaningful use of data to support quality of care and CQI; and corporate support functions and structures.

**Conclusion:**

The framework should be useful for guiding development and implementation of barrier-driven, tailored interventions for primary health care service delivery and policy contexts, and for guiding further research. While specific strategies to improve the quality of preventive care need to be tailored to local context, these findings reinforce the requirement for multi-level action across the system. The framework and findings may be useful for similar purposes in other parts of the world, with appropriate attention to context in different locations.

**Electronic supplementary material:**

The online version of this article (doi:10.1186/s12992-017-0267-z) contains supplementary material, which is available to authorized users.

## Background

As with other colonised populations worldwide, Aboriginal and Torres Strait Islander Australians (hereafter referred to respectfully as Indigenous Australians) experience poorer health outcomes and shorter life expectancy compared with non-Indigenous Australians [1–4]. Providing equitable access to primary health care (PHC) is a continuing challenge, despite a universal health insurance scheme (Medicare^1^) and the funding of community-controlled and government-managed health services specifically designed to meet the health needs of Indigenous Australians (in addition to private general practices) [3, 5]. Colonisation, social determinants, and discrimination are important factors in these inequities [4, 5]. Potentially preventable chronic diseases are the greatest contributor to the difference in health status between Indigenous and non-Indigenous Australians [1]. The role of preventive care in the early detection and management of chronic disease is widely recognised [1, 6, 7].

Consistent with international trends to improve the delivery of preventive health services the Australian Government has introduced policy initiatives to enhance the delivery of preventive care for Indigenous people. Medicare-funded Indigenous-specific health assessments were introduced in 1999 and progressive expansion of these services has resulted in a substantial increase in the delivery of assessments over the past 15 years [8–10]. Unfortunately, these efforts have had no clear effect on improving mortality and morbidity [11, 12] and follow-up from health assessments has been disproportionally low [8, 9]. This has called into question the effectiveness of health assessments as a way to ensure Indigenous Australians get preventive health services [8, 13]. Concerns have been raised that health assessments may not be reaching those most in need, thereby reducing the potential benefits at a population level [8, 9, 11–13]. Consistent with earlier studies [14, 15], a 2016 assessment of the delivery of recommended preventive care for Indigenous Australians found substantial deficiencies in the delivery of recommended care, wide variation in the delivery of service items and a lack of follow-up of abnormal clinical findings [16]. Despite efforts to promote evidence-based preventive care at the PHC level [6] and policy initiatives such as preventive health assessments, delivery of guide-line recommended preventive health care to Indigenous Australians remains suboptimal.

Evidence-practice gaps across many health centres are often due to failures or weaknesses of the wider health system [17, 18]. Large-scale improvement in preventive PHC delivery could be achieved through identifying priority evidence-practice gaps in care and using the information to inform action across the health system [17]. Actions or interventions designed to address known barriers to quality care are more likely to produce change, however few interventions are based on a systematic assessment of barriers [19, 20]. The need for further development of methods to identify barriers and design interventions to address these barriers has been identified [21, 22].

A ‘co-creation approach’ involving researchers, clinicians, administrators, community members and policy makers has been advocated for identifying priorities and driving improvements in care [18, 22, 23]. The value of diverse stakeholder perspectives in improving Indigenous primary health care (PHC) has been established [24]. We drew on this evidence to design a mixed-methods study to engage diverse PHC stakeholders in interpreting aggregated CQI data on preventive care.

The aim of this paper is to describe stakeholder identified priority evidence-practice gaps, stakeholder perceptions of barriers and enablers and suggested strategies for improving preventive care. We use this co-created information to develop a causal pathway diagram, presented as a framework of key factors (or drivers) to improve the delivery of guideline recommended preventive care. This paper contributes to the identification of ways to improve large-scale delivery of preventive health services, thereby reducing health inequalities in access to quality preventive care.

## Methods

Developed in 2013, the “Engaging Stakeholders in Identifying Priority Evidence-Practice Gaps and Strategies for Improvement in PHC” (ESP) project brought together the concept of knowledge co-creation [22, 23] and evidence on how to achieve large-scale change [18, 24]. It aimed to engage a wide range of stakeholders in using aggregated continuous quality improvement (CQI) data to identify priority gaps in care, barriers or enablers and strategies for improvement. The ESP Project methods and theoretical base are described in detail elsewhere [18].

### Clinical audit and systems assessment

The ESP Project has drawn on CQI data provided by health centres to the Audit and Best Practice for Chronic Disease (ABCD) National Research Partnership, a wide-scale, research-based CQI initiative (2010–2014) [25, 26]. Over 17,000 client records in 137 Indigenous PHC centres were audited for preventive health practices and included in the analysis.

As part of their routine CQI activities, participating health centres performed annual audits of client medical records to determine whether recommended preventive service items were documented as delivered in the previous 24 months [27]. The audit tool and parameters of the outcomes measures were developed by an expert working group and based on evidence and best practice guidelines. To be eligible for inclusion in the audit, a client must: be between 15 and 55 years; resident in the community for at least 6 months; have no diagnosis of diabetes, hypertension, coronary heart disease, chronic heart failure, rheumatic heart disease or chronic kidney disease; not be pregnant or not less than 6 weeks postpartum at the time of audit; and have at least one attendance at the PHC in the previous 24 months. The audit protocol included sampling guidelines to generate a sample likely to reflect the general population of clients. A structured process to assess the organisational systems of the PHC was conducted using the Systems Assessment Tool (SAT) [28].

### Ethics

The study was approved by human research ethics committees in the relevant states and territories [25]. All participants in the ESP Project surveys provided individual informed consent.

### Project Phases

The project comprised three phases: 1) identifying priority evidence-practice gaps, 2) identifying barriers and enablers to addressing these gaps, 3) data synthesis for development of a framework of drivers and strategies. These phases are described in more detail below. For phases 1 and 2 (the ESP Project), we targeted stakeholders representing diverse roles, PHC settings and organisations who had been identified as having an interest in Indigenous PHC service delivery (including those who participated in the CQI audits), management, research and policy. To enable engagement of those less likely to provide individual responses, we encouraged responses from facilitated group discussions. Some group responses indicated large numbers of participants. Groups reported to be larger than 100 were recorded as 20 individuals to more realistically reflect likely numbers of active contributors. The estimated number of people providing input may therefore be conservative. Networks established over the years of the ABCD National Research Partnership were used to develop circulation lists for the ESP reports and surveys. A snowballing distribution technique was utilised, encouraging people to forward reports and surveys through their professional networks.

#### Phase 1 – identifying priority evidence-practice gaps in preventive care

During this phase we presented a report of cross-sectional clinical preventive care audit and systems assessment data (2012–2014; 3571 clinical audit records and 71 systems’ assessments from 95 health centres).

The research team developed a preliminary set of priorities using the following criteria: aspects of care that were recorded at low levels; aspects of care where there was wide variation in recorded delivery; or organisational systems that were relatively less developed (based on SAT data) (Additional file 1: Table S1). Through an online survey we asked stakeholders whether the preliminary priorities accord with their experience, to rank the priorities by perceived importance; and to determine if other priorities should be included.

#### Phase 2 Identifying barriers and enablers and strategies for improvement

**Fig. 1:**
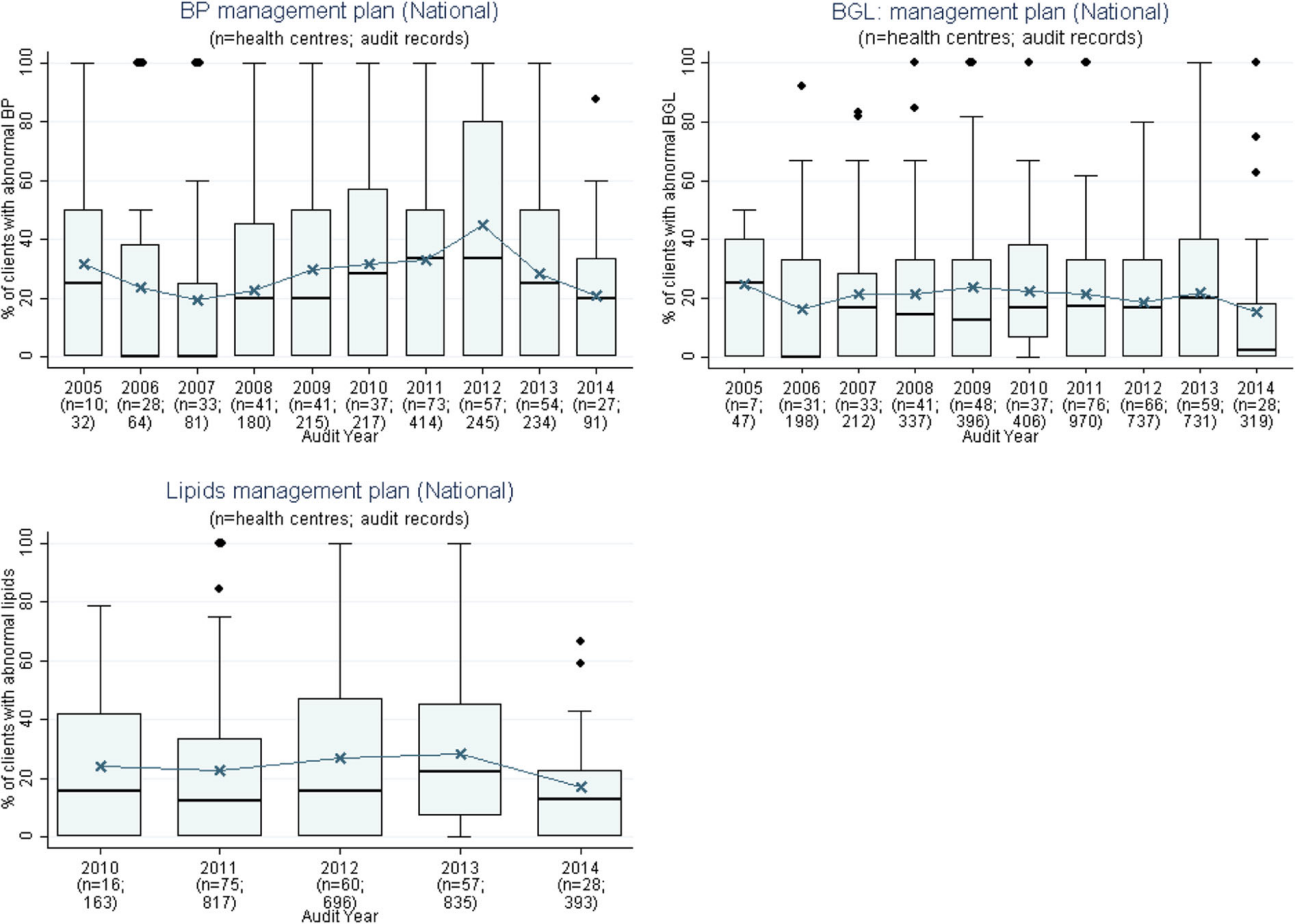
Mean health centre record of plan for follow-up of abnormal blood pressure, blood glucose level and lipid profile, by audit year. Note: Lipid test was introduced into the preventive health audit tool in August 2010

**Fig. 2:**
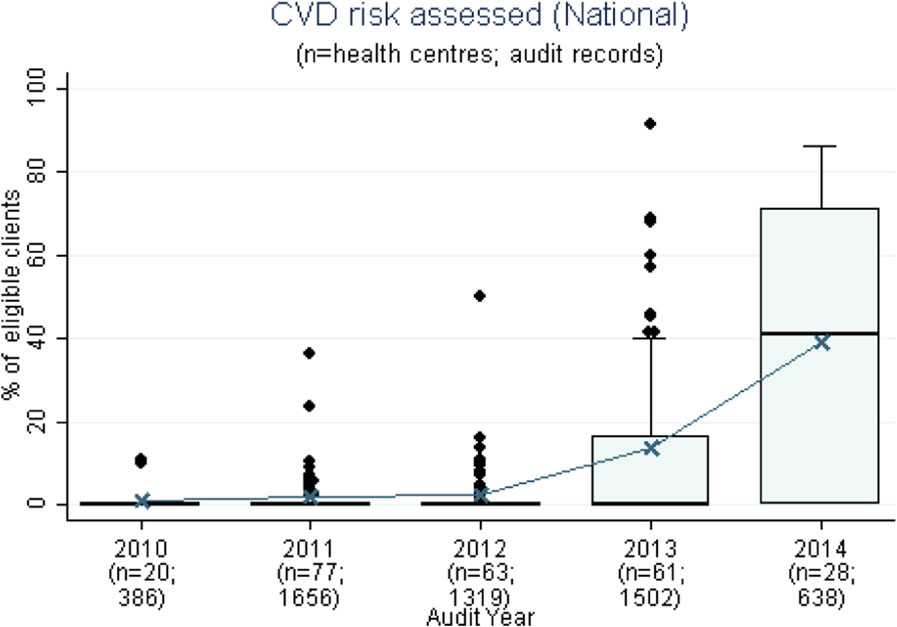
Mean health centre recording of cardiovascular risk assessment, by audit year. Note: This item was introduced into the preventive health audit tool in August 2010. According to best practice guidelines, clients eligible for absolute cardiovascular risk assessment if: Indigenous, ≥35 years of age and not a resident of the Northern Territory; or Indigenous, ≥20 years of age and a resident of the Northern Territory; or non-Indigenous and 45 years and over

**Fig. 3:**
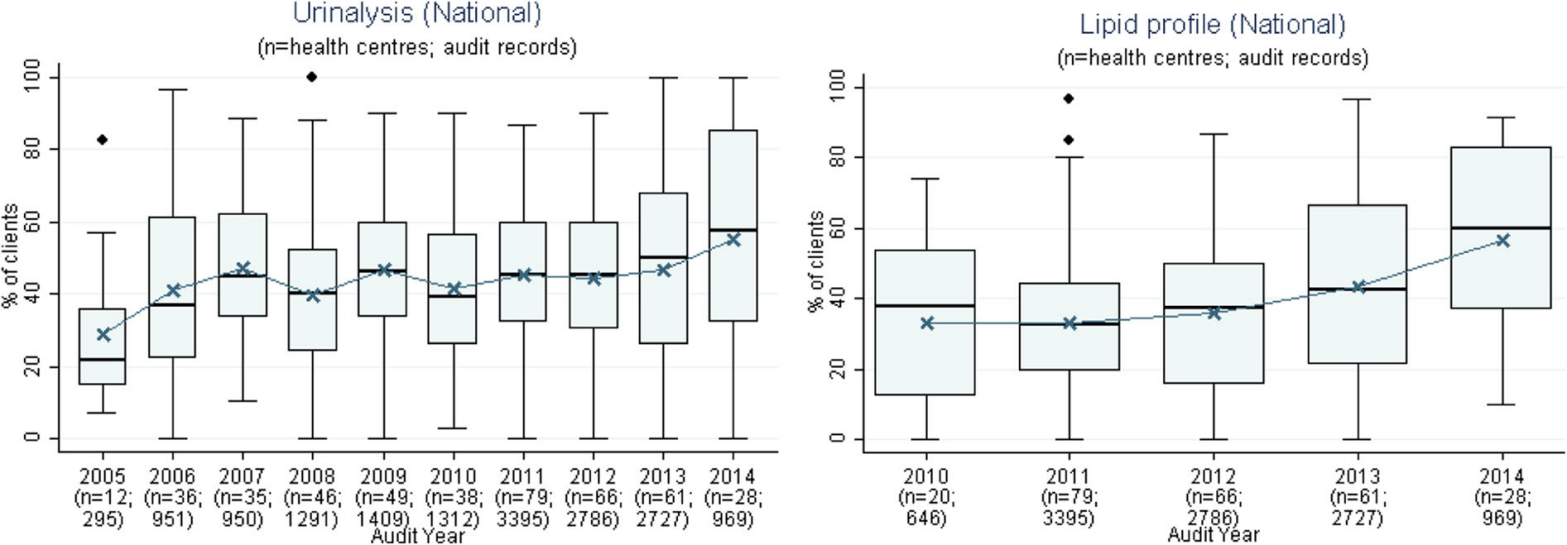
Mean health centre record of urinalysis and lipid profile, by audit year. Note: Lipid test was introduced into the preventive health audit tool in August 2010

**Fig. 4:**
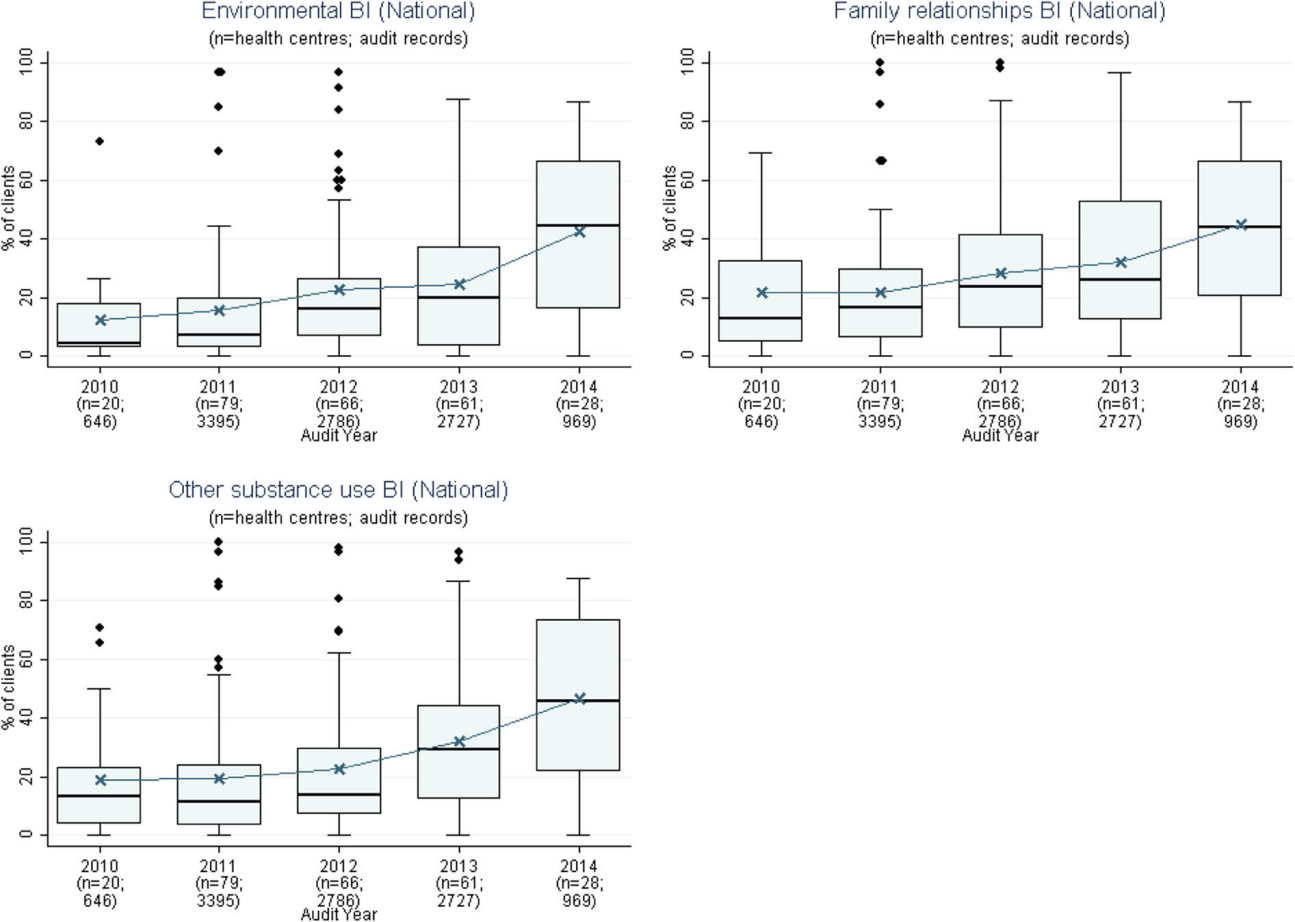
Mean health centre percentage of clients with record of enquiry regarding environmental and living conditions, family relationships and other substance use, by audit year. Note: These items were introduced into the preventive health audit tool in August 2010

**Fig. 5:**
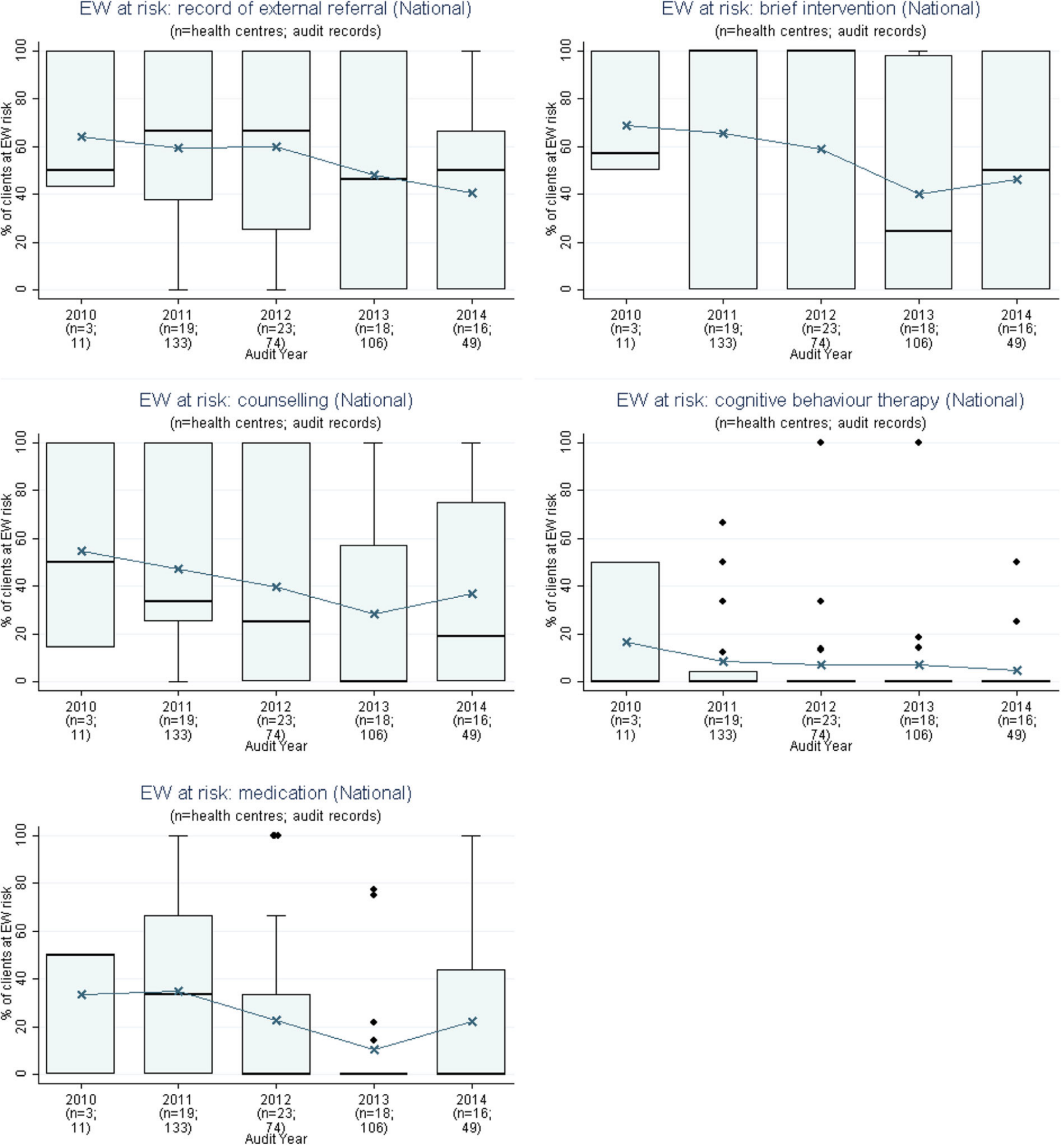
Mean health centre percentage of clients with a record of emotional wellbeing (EW) follow-up action if identified at risk using a standard tool, by audit year. Note: These indicators were introduced in the audit tool in August 2010 and apply to those clients that had a record of being at risk of an emotional wellbeing issue

**Fig. 6:**
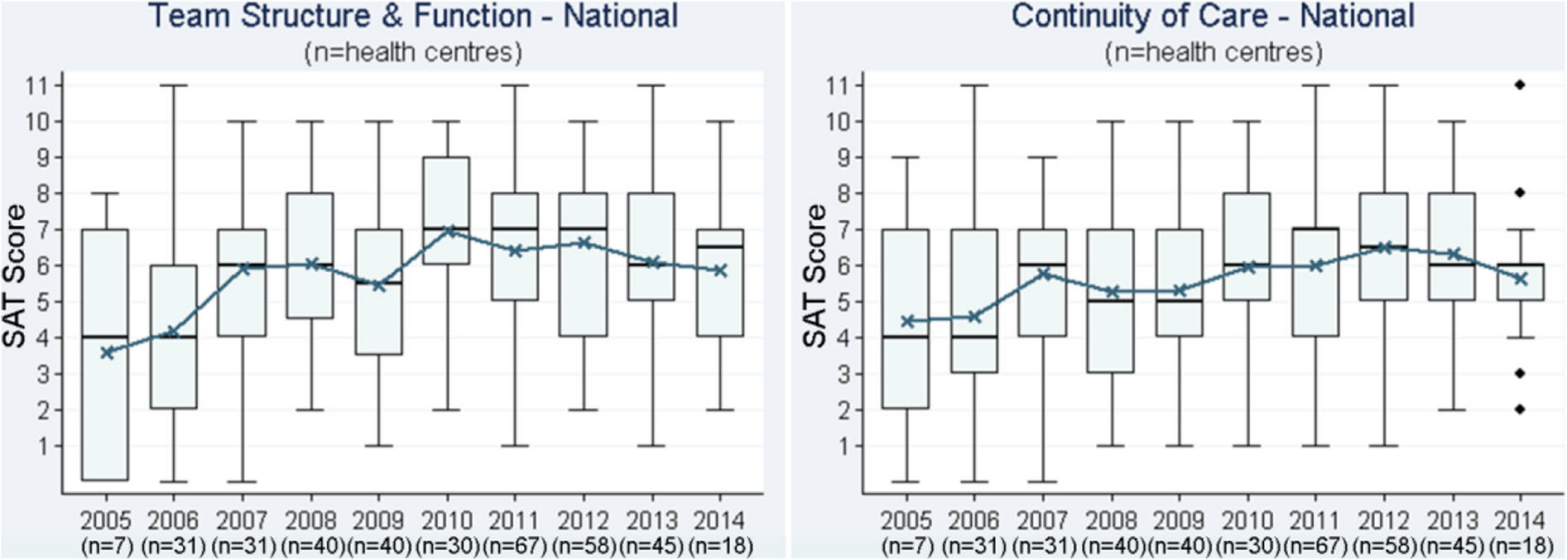
Team structure and function and continuity of care component scores, by audit year

**Fig. 7:**
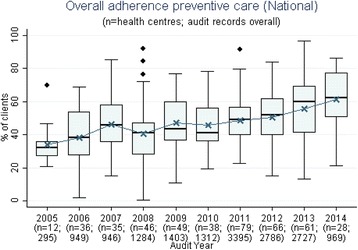
Mean health centre overall service delivery to well clients. Note: Overall preventive care service delivery composite figure includes: weight, waist circumference, blood pressure, urinalysis, blood glucose level, sexually transmitted infections (gonorrhoea and chlamydia; syphilis), pap smear, oral health, nutrition, physical activity, smoking and alcohol status recorded, brief intervention if smoker and/or high risk alcohol user

We presented a report of the phase 1 findings and trend audit data (2005–2014) from 137 health centres (17,108 clinical audit records and 367 system assessments) that examined trends over time for priority evidence-practice gaps as agreed upon in Phase 1 (Figs. 1, 2, 3, 4, 5, 6, and 7).

Through an online survey, we asked respondents to focus on the trend data and their experience in PHC to a) identify barriers and enablers to improvement and b) new or existing strategies to address the gaps. The survey drew on national and international evidence on health system and staff attributes (domains) that may present obstacles to improvement, such as insufficient finances and resources, lack of patient centred-care and systematic quality improvement. The survey instrument has been published elsewhere [29]. As a member checking process, we distributed the draft final report and invited stakeholder feedback on whether we had accurately captured their views.

#### Phase 3 Data synthesis for development of framework

Drawing on the survey data provided in the previous phase on the barriers and enablers for addressing the gaps in preventive care, the authors undertook an iterative process to develop a framework for improvement as follows: (1) multiple readings of the survey data and an initial assessment of the emerging barriers and enablers to addressing gaps in care were undertaken by the lead author (JB), using an organising matrix of ‘health system’ or ‘staff attributes’; (2) drawing on this thematic analysis the lead author produced a framework or ‘driver diagram’, a diagrammatic quality improvement tool used to position identified barriers and enablers within causal pathways (referred to as key drivers) [30]; (3) strategies for improvement identified by stakeholders were aligned with the relevant drivers; (4) the driver diagram was reviewed and refined over several iterations by all authors. The authors collectively possess a depth of experience in preventive health provision to Indigenous people, as clinicians, program leaders, policy makers and researchers.

## Results

Approximately 152 individuals participated in the online surveys for phases 1 and 2, either as individuals or members of a group (Table 1). Organisations represented included community-controlled and government health services, support and policy organisations, and research institutions. Respondents included nurses, senior managers, CQI facilitators, researchers, Aboriginal Health Practitioners (AHP), policy officers and doctors (Table 1).

**Table 1:** Survey responses for the preventive health ESP Project phases

	Phase 1 – Identifying priority evidence-practice gaps		Phase 2 – Barriers and enablers to and strategies for improvement	
	Individual responses	Group responses^a^	Individual responses	Group responses^a^
Number of responses	15	4 (on behalf of an estimated 62 people)	3	4 (on behalf of an estimated 70 people)
Jurisdictions of interest for respondents^b^				
National	1		1	
NSW	0		1	
SA	1		0	
Queensland	5		1	
WA	3		1	
NT	6		1	
Victoria	4		2	
Rurality of population group to which responses relate^b^				
Urban	7		6	
Regional	8		4	
Remote	13		4	
Number of group responses to question about Indigenous status				
Majority (more than half)		2		2
Minority (less than half)		2		2
Number of individual responses identified as Indigenous				
Indigenous	2		1	
Non Indigenous	13		2	
Position types^b^				
Nurse, doctor or specialist	6	4	0	4
Middle or senior management, board member	3	3	0	6
CQI facilitator	1	1	1	1
Policy officer	0	0	0	1
Aboriginal and/or Torres Strait Islander practitioner	0	2	0	4
Research/Academic	1	1	0	0
Other	4	1	2	1
Organisation types^b^				
Community-controlled peak body/health centre	6	2	0	1
Government health department/health centre	8	1	1	0
Medicare Local or PHC Network	0	0	0	2
University/Research organisation	0	1	1	0
Other	1	1	1	1

^a^Some groups indicated large numbers – considerably more than 20 and in some instances more than 100. It was not clear how many individuals provided actual input. For the purpose of estimating the numbers who provided actual input we have used a figure of 20 individuals for groups that were reported to be larger than 20. The estimated number of people providing input may therefore be conservative

^b^Numbers may not tally with total number of respondents, as respondents were able to select multiple answers

### Priority evidence-practice gaps in preventive care identified by stakeholders

Approximately 77 people (15 individuals and four group responses on behalf of 62 people) participated in phase 1. Characteristics of the health centres providing CQI data are presented in Table 2. Overall, 79% of health centres were in remote locations and 73% were government managed.

**Table 2:** Characteristics of participating health centres and patients whose records were audited between 2005 & 2014 (number & %)

		2005		2006		2007		2008		2009		2010		2011		2012		2013		2014		Overall	
Primary Health Care Centres		12		36		35		46		50		38		79		66		61		28		137	
		N	%	N	%	N	%	N	%	N	%	N	%	N	%	N	%	N	%	N	%	N	%
Location	Urban	0	0	4	11	0	0	4	9	1	2	1	3	4	5	5	7.58	1	2	5	18	11	8
	Regional	5	42	10	28	6	17	9	20	5	10	2	5	5	6	5	7.58	5	8	3	11	18	13
	Remote	7	58	22	61	29	83	33	72	44	88	35	92	70	89	56	84.85	55	90	20	71	108	79
Governance	Community-controlled	8	67	25	69	22	63	22	48	20	40	11	29	13	16	7	11	7	11	7	25	37	27
	Government	4	33	11	31	13	37	24	52	30	60	27	71	66	84	59	89	54	89	21	75	100	73
Number of audited records		**358**		**1029**		**1015**		**1420**		**1554**		**1416**		**3557**		**2893**		**2875**		**991**		**17,108**	
Age (mean & range)		32 (15–55)		32 (15–63)		32 (15–61)		34 (15–65)		35 (15–65)		35 (15–65)		33 (15–65)		33 (15–65)		33 (15–65)		31(15–65)		33 (15–65)	
Sex	Male	176	49	504	49	503	50	713	50	786	51	720	51	1836	52	1473	51	1433	50	507	51	8651	51
Indigenous status	Yes	250	70	962	93	907	89	1286	91	1476	95	1183	84	3015	85	2447	85	2485	86.43	897	91	14,908	87
Attended within last 24 months		295	82	958	93	952	94	1293	91	1454	94	1312	93	3395	95	2786	96	2727	95	969	98	16,141	94
Reason for last attendance	Well Person’s Check	8	2	118	11.5	107	11	133	9	104	7	149	10	381	11	375	13	423	15	120	12	1918	11
	Acute care	151	42	509	49.5	528	52	717	50.5	750	48	657	46	1780	50	1356	47	1418	49	462	47	8328	49
	Other	186	52	339	33	370	36	559	39.5	688	44	531	38	1234	34	1055	36	886	31	387	39	6235	36
Not recorded		13	4	63	6	10	1	11	1	12	1	79	6	162	5	107	4	148	5	22	2	627	4
Profession patient first seen by	AHP	53	15	269	26	195	19	335	23.59	293	19	265	18.7	674	19	617	21	534	18.6	154	16	3389	20
	Nurse	127	35	362	35	473	47	582	40.99	774	50	610	43.1	1899	53	1494	52	1458	50.7	579	58	8358	49
	GP	71	20	227	22	245	24	329	23.17	272	18	292	20.6	573	16	514	18	584	20.3	188	19	3295	19
	Other	15	4	39	4	40	4	79	5.56	76	4	71	5	132	4	75	2	121	4.2	48	5	696	4
	Not recorded	92	26	132	13	62	6	95	6.69	139	9	178	12.6	279	8	193	7	178	6.2	22	2	1370	8

*CQI* continuous quality improvement, *GP* general practitioner, *AHP* Aboriginal health practitioner. ‘Other’ *reason for last attendance* includes appointments for mental illness, immunisation, antenatal and sexual health etc. ‘Other’ *profession patient first seen by* includes specialists and allied health professionals

The aggregated CQI data showed that some aspects of preventive care were being provided and documented at high levels by health centres. The aspects of care in which there was relatively better recording included up-to-date health summaries and immunisation records, measurement of weight, blood pressure, pulse rate and rhythm, delivery of brief interventions for clients identified as using alcohol at high risk levels, and recording of Medicare numbers. However, wide variation between health centres was evident in almost all aspects of preventive care.

Stakeholders identified seven evidence-practice gaps as priorities for improvement. These are presented in order of perceived priority:

- Follow-up of clients with abnormal blood pressure, blood glucose levels and lipid profile (Fig. 1)
- Completing absolute cardiovascular risk assessments (Fig. 2)
- Recording of urinalysis (Fig. 3)
- Recording of lipid profiles (Fig. 3)
- Recording of enquiry about environmental & living conditions, family relationships and substance abuse (Fig. 4)
- Providing appropriate support and follow-up for clients identified as being at risk with respect to emotional wellbeing (Fig. 5)
- Strengthening ‘team structure and function’ and ‘continuity of care’ (Fig. 6)

Although delivery of care relating to the identified evidence-practice gaps in preventive care was low, there was evidence of improvement over time (Fig. 7).

Stakeholder feedback on the priority evidence-practice gaps highlighted the importance of continuing attention to holistic care, and of ensuring that focus on specific indicators does not detract from the importance of providing high quality care across the scope of best practice. A majority of respondents (76%) considered improvement across all health centres as a priority rather than prioritising action for health centres performing at relatively lower levels.

### Key drivers and strategies for addressing identified gaps in preventive care

**Fig. 8:**
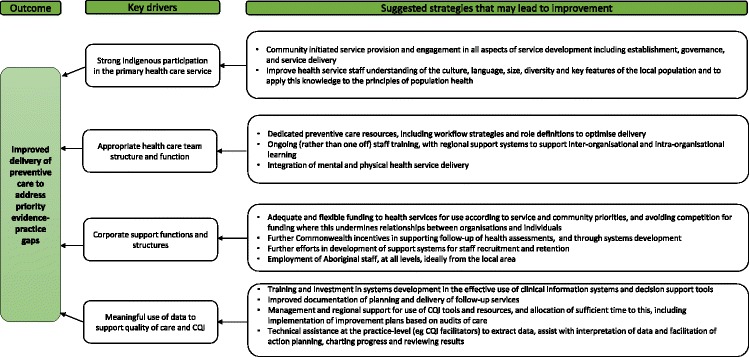
Drivers of high quality preventive care and suggested strategies for addressing identified evidence-practice gaps

Approximately 73 stakeholders (three individuals and four group responses on behalf of approximately 70 people) identified barriers and enablers to addressing the identified evidence-practice gaps. The analysis of the barriers and enablers identified four key drivers for addressing priority evidence-practice gaps, each with suggested strategies (Fig. 8). The identified drivers are interdependent and some findings are relevant to more than one driver. We have therefore described the findings according to the predominant driver, as follows.

#### Strong Indigenous participation in the primary health care service

Most respondents identified a need to improve links between health services and communities, and enhance health literacy. The critical brokering role of AHPs in increasing access to, acceptability of and trust in the health service was acknowledged. Audit data indicated that only 20% of clients were recorded as seeing an AHP as the first point of contact when attending the health service (Table 2). Respondents commented on the important role of Indigenous staff in engaging clients in their own health, through preventive health assessments and follow-up consultations. This was linked to an identified need for more Indigenous staff at all levels of the PHC service, ideally members of the local community. High staff turnover was viewed as a barrier to developing and maintaining links between health services and communities. Respondents perceived that local orientation to the culture, language, and diversity of their service populations was generally lacking, as well as skills in applying this knowledge to reflect the principles of population health.

#### Appropriate primary health care team structure and function

Respondents noted the need for adequate staffing levels to allow the time required to deliver preventive health services. High demand for acute care was highlighted as a barrier, while dedicated preventive care resources, workflow strategies to enable delivery of preventive care and clear role definitions were viewed as enablers to improving the delivery of preventive care. Responses indicated that at times staff have trouble focusing their attention to provide best practice care in preventive health due to competing demands. This is borne out in audit data, which show that 48% of clients last attended their health service for acute care and only 11% for a preventive health assessment (Table 2). Despite competing demand on staff time and its negative impact on attending to preventive health care, health service staff were generally perceived as knowing the content and objectives of best practice preventive health care delivery.

Stakeholders identified the need for close collaboration between health promotion staff and clinicians within PHC teams and health promotion activities linked to local community needs. Further, it was felt there should be a greater focus on ensuring that all programs and service delivery are based on the needs and aspirations of Indigenous communities, and that care provision is respectful of and responsive to individual preferences, needs and values.

Indigenous PHC services are characterised by high staff turnover, particularly in remote locations. Within this environment, ongoing (rather than one-off) staff training, supported by regional systems to foster inter-organisational and intra-organisational learning, was considered an enabler to the delivery of preventive care. Survey respondents specifically identified the need for training in team work, patient-centred care and self-management support, cultural competence and cultural safety and understanding of the impact of social determinants on health. Integration of services (rather than merely co-location) for mental health and wellbeing care and physical health was suggested to further support preventive care.

#### Corporate support functions and structures

##### Financing and resources to support preventive care

Medicare-funded Indigenous-specific preventive health assessments [10] were acknowledged as a useful funding stream. Stakeholders specified a need for increased funding to deliver follow-up services relating to issues identified in health assessments, including system improvements to support follow-up. Further work was suggested to avoid competition for funding where this undermines relationships between organisations and individuals, and to build a culture of partnerships and collaboration between service providers. Stakeholders identified the need for flexible funding to enable services to be responsive to community needs and target prevention activities accordingly.

##### Effective strategies for recruitment and retention of staff

Workforce issues were frequently identified as impacting on the effectiveness of preventive care, including high staff turnover and skill mix. Development of regional support systems for recruiting and retaining staff, especially AHPs, was identified. Recruiting Indigenous staff (particularly local staff) and ensuring effective support, was seen as critical to ensuring the community-health service connections and the provision of a culturally appropriate service that is perceived to be accessible by the community. Improved role definition and clarity based on the identified strengths of the AHP workforce and access to adequate numbers and types of staff for follow-up services were identified as important enablers.

#### Meaningful use of data to support high quality care delivery and continuous quality improvement processes

Further investment is required in systems development and practitioner training in the effective use of clinical information systems and decision support tools, for example recall and reminder systems and cardiovascular risk assessment calculators. Improved documentation of care provided to clients and efficient upload of test results in the correct fields of information systems were deemed necessary to avoid duplication of efforts and support team-based care.

Most respondents agreed on the need for further management support to enable staff in health services to use CQI tools and resources, and to allocate sufficient time to CQI activities, including the implementation of improvement plans based on audits. Technical assistance at the practice-level (for example CQI facilitators) to extract data, assist with interpretation and facilitation of action plans, chart progress and review results was seen to enhance meaningful use of data to support care.

## Discussion

Improving the delivery of preventive care for Indigenous Australians is crucial to closing the health gap between Indigenous and non-Indigenous Australians. Current delivery of preventive care for Indigenous Australians is suboptimal with wide variation in the delivery of recommended preventive care between health centres. This study engaged a wide range of stakeholders in using the most comprehensive data set of its kind currently available in Australia to identify priority evidence-practice gaps in preventive healthcare across 137 health centres that serve Indigenous Australians, and the key drivers to address these gaps.

The identified gaps in preventive care included: follow-up of abnormal results; completing cardiovascular risk assessments; timely recording of test results; recording enquiries about living conditions, family relationships and substance abuse; provision of support and follow-up for those at risk with respect to emotional wellbeing. System refinements that improve ‘team structure and function’ and ‘continuity of care’ were also identified priorities. This collaboratively created knowledge has been used to develop a framework of drivers for improving the delivery of care. They include strong Indigenous participation in the PHC service, appropriate team structure and function to support preventive care, meaningful use of data to support high quality care delivery and CQI, and effective corporate support functions and structures.

Follow-up of patients after a health assessment has been reported as low in Australia and elsewhere [8, 9]. Our study identified follow-up of abnormal results as the most pressing priority for improvement, reflecting similar findings of other studies [8, 31]. Participants in our study identified drivers of quality preventive care at different health service levels. As seen elsewhere in the world, identifying the most important drivers for change is difficult [19]. Drivers for achieving the health benefits of screening and assessments at health centre level included accurate documenting of care in patient records as a way of enabling timely care provision, a team-based approach, and avoiding over-servicing (e.g. unnecessary repeating of laboratory investigations). A number of the priorities and drivers identified are beyond the influence or control of individual health centres and services, and require stronger engagement from higher level management and policy makers. For example, improving the quality of social and emotional well-being care requires a model of integrated care that addresses social determinants through inter-sectoral and regional collaborations in service delivery and human resource management.

Identified drivers for improved preventive care are consistent with national [5, 8, 16, 32–35] and international literature on barriers and enablers to care [36, 37]. Our deliberate strategy of seeking data interpretation and input from those with tacit and professional knowledge of delivering preventive care to Indigenous clients and communities helped to ensure that the strategies suggested are consistent with the important principle of providing culturally safe, patient-centred care [34]. Culturally unsafe PHC environments are recognised as a barrier to care access for Indigenous Australians and for Indigenous populations in other countries around the world [4, 5].

Key emerging challenges will be implementing a multi-sectoral and systems-wide approach that goes beyond health to involve other service agencies [38, 39] and refocusing the attention of funders towards preventive health [40]. Taking high level action to meet these challenges in the Indigenous PHC sector would no doubt have benefits for the wider Australian population, in which little or no progress is being made in preventing and controlling risk factors for chronic disease (with the exception of tobacco control) [40].

### Strengths and limitations

A particular strength of our study was the measurement of a broad range of service delivery indicators for preventive health care delivery based on best practice clinical guidelines, and the engagement of diverse healthcare stakeholders to interpret the aggregated CQI data. Strengths of the analysis include the iterative process of stakeholder engagement to develop a framework of drivers and strategies to improve preventive care. Individuals and groups could choose to participate in any or all ESP project phases. The ESP project has relied, in part, on stakeholders sending reports to others. Thus, a limitation of the study is that it has not been possible to accurately measure the reach of report dissemination and survey response rates. Limitations include voluntary enrolment in the ABCD CQI program, with uncertainty in the generalisability of the findings. Audit data are based on recorded delivery of services, which generally underestimate actual service delivery. The findings represent feedback from a diverse range of stakeholders working in Indigenous PHC service delivery, policy and research. However, they primarily represent the views of the Northern Territory and Queensland jurisdictions and remote and rural contexts.

## Conclusion

The framework presented offers opportunities for regional-level support organisations and policy makers to develop barrier-driven, tailored interventions to improve the delivery of preventive care for Indigenous Australians. Such system-level action should be developed with a deep understanding of the holistic nature of Indigenous Australians wellbeing beyond just physical health (including healthy connections to culture, community and country), of the impact of Australian colonial history on Indigenous Australians, and of how social systems – including the health system - should be shaped to meet the needs of Indigenous Australians.

The framework should assist those developing PHC policy, interventions and training to develop tailored interventions to improve health outcomes, and guide further research. While specific strategies to improve the quality of preventive care need to be tailored to local context, these findings reinforce the requirement for multi-level action across the system. The framework and findings may be useful for similar purposes in other parts of the world, with appropriate attention to context in different locations.

## Additional file

Additional file 1: Identified preliminary priority evidence-practice gaps [26]. (DOCX 22 kb)

## Abbreviations

ABCD: Audit and best practice for chronic disease
AHP: Aboriginal health practitioner
CQI: Continuous quality improvement
ESP: Engaging stakeholders in identifying priority evidence-practice gaps and strategies for improvement in primary health care
PHC: Primary health care
SAT: Systems assessment tool

## Footnotes

1 Medicare is Australia’s universal health insurance scheme. Medicare provides access to a range of medical services, lower cost prescriptions and free care as a public patient in a public hospital.
