# Supplementary material for: Improving preventive health care in Aboriginal and Torres Strait Islander primary care settings
Source: Global Health. 2017 Jul 14;13:48. doi: 10.1186/s12992-017-0267-z (PMC5512740; doi:10.1186/s12992-017-0267-z)
Supplement: Additional file 1: — Identified preliminary priority evidence-practice gaps [26]. (DOCX 22 kb) [file 12992_2017_267_MOESM1_ESM.docx]

**Additional file**

**Additional file 1*:Table S 1: Identified preliminary priority evidence-practice gaps [26]***

| **Clinical history and observations** | *History:*  Hearing problems  Discussion of sexual and reproductive healthcare  Continence  Alcohol use  Tobacco status  Other substance use  Emotional well-being assessment  Environmental and living conditions  Family relationships | *Examination:*  BMI, waist circumference  Urinalysis  Visual acuity  Oral health  Ear examination findings & hearing problems |
| --- | --- | --- |
| **Laboratory and radiology testing** | Lipid profile  Pap smear  Mammography | |
| **Synthesis of information** | Absolute cardiovascular risk assessment  Completion of a health check | |
| **Follow-up and clinical care** | Follow-up of abnormal BP, BGL and lipid profile  Provision of advice on nutrition and physical activity  Appropriate support and follow-up for clients at risk of social and emotional problems | |
| **Health systems** | Linkages to community and other health service  Self-management support  Team structure and function  Continuity of care | |
